# The power of the ‘universal’: caste and missionary medical discourses of alcoholism in the Telugu print sphere, 1900–1940

**DOI:** 10.1017/mdh.2023.30

**Published:** 2023-10

**Authors:** Tarangini Sriraman

**Affiliations:** Goldsmiths University of London, History, 8 Lewisham Way, London

**Keywords:** missionary, medical discourses, alcohol, caste, Telugu, toddy, arrack

## Abstract

This article explores missionary medical discourses in three Telugu journals published in the early twentieth century, to analyse how caste pivoted denunciations of alcohol, especially toddy and arrack, in the Madras Presidency and the Hyderabad state. It argues that one women’s missionary journal, *Vivekavathi*, deployed medical knowledge to formulate subtle and occasionally explicit condemnations of toddy and arrack as unclean and unhealthy substances. The journal relied on universal medical and missionary, British and American knowledge frameworks to mark out Dalits and other marginalised castes as consumers of these local beverages. This stigma was conjured through medical narratives of marginalised castes as lacking in the knowledge of alcohol’s relation to digestion, toddy’s role in ruining maternal and child nutrition, the unhygienic environment of arrack shops and their propensity to ‘alcoholism’. However, this article also traces counter-caste voices who too invoked ‘the power of the universal’ to dispel caste stigma against marginalised castes. While both sets of voices deployed medical ‘enslavement’ to alcohol as an interpretive move, they differed in their social imperatives and political imaginaries, defined in caste terms. This article explores a third set of implications of the term ‘universal’ by analysing global medico-missionary narratives of alcohol in two other Telugu journals. On a methodological plane, this article also pushes for a hybrid reading of what counts for ‘scientific instruction’, where hymns, catechisms, parables and allegories are considered alongside conventional scientific experiments. In that sense, it upholds vernacular missionary publications as an invaluable resource for the social history of medicine.

They have also found out that alcohol intake disrupts the process of digestion. Dr Monroe (sic) from Hull has carried out an experiment. He diced the beef into small pieces, extracted its gastric juices, and divided two portions equally into three parts. He then added water into one container*, toddy* into another, and *arrack* into yet another container. He created a natural stomach situation for those containers at 100**°** and began to observe the changes they underwent (emphasis mine).^1^

Toddy *enslaves you to debt*
It is the origin of dangers
It is the biggest cause of mistakes
It is the instrument of pain
On account of drinking toddy,
Your hands and feet give up on you
Your throat and passages
Are afflicted by a stench
When you drink arrack
You are causing diseases
When you smoke cigarettes
Your chest and heart burn (emphasis mine).^2^

They taste the local and foreign alcohols first, feel a growing attachment the second time, and *become slaves to alcohol* the third time. Compared to the other intoxicants we have discussed above, alcohol is much more dangerous. It disrupts the flow of the blood, impacts liver function, burns the heart, and kills you a little day by day. It decreases your life expectancy. People die suddenly because of their drinking habits (emphasis mine).^3^

This article delves into the politics of missionary medical temperance in the Telugu print world of the early twentieth century Madras Presidency, especially between 1900 and 1940. In so doing, it is invested in exploring how medical temperance writings in Telugu missionary publications – that touch on the toxicity of alcohol and its harm to human digestion, diet and nutrition – discern the subject of caste without always naming it. The invocations of caste feature in a temperance world brimming with overlapping medical, social and cultural descriptions of toddy and arrack. Toddy refers to a fermented beverage common to South India and, depending on the region, is tapped from the palmyra, coconut, date or sago palm tree. Arrack is typically spirit distilled from toddy or grain, ‘cane sugar, molasses or jaggari’.^4^

Instead of situating alcohol in colonial South India within nationalist and subaltern frameworks of anti-drink movements, this article locates it within two sites, the social history of medicine and a rare historical genre, namely, the vernacular missionary journal.^5^ While caste has pivoted the critique of nationalist and missionary discourses of alcohol, it has not featured in a systematic analysis of medical histories of alcohol in colonial India.

The three early-twentieth-century Telugu missionary journals discussed in this article are *Vivekavathi*, *The Telugu Baptist* and *The United Church Herald* (*UCH*) (Figures 1–3). While none of them was a health journal, they nonetheless provided health-driven commentaries on alcohol. *Vivekavathi* authors especially presented excerpts, summaries and interpretations of experiments and medical findings by scientists, chemists and physicians writing on alcohol in the UK and America. This article argues that what was presented as medical and scientific knowledge carried often subtle and occasionally explicit condemnations of toddy and arrack as unclean and unhealthy substances. *Vivekavathi* associated these beverages with Dalit and non-Dalit marginalised communities. This article simultaneously argues that the same medical debates and discussions in *Vivekavathi* produced subtle counter-caste voices that sought to banish this stigma from the lives of marginalised castes.

**Figure 1–3:**
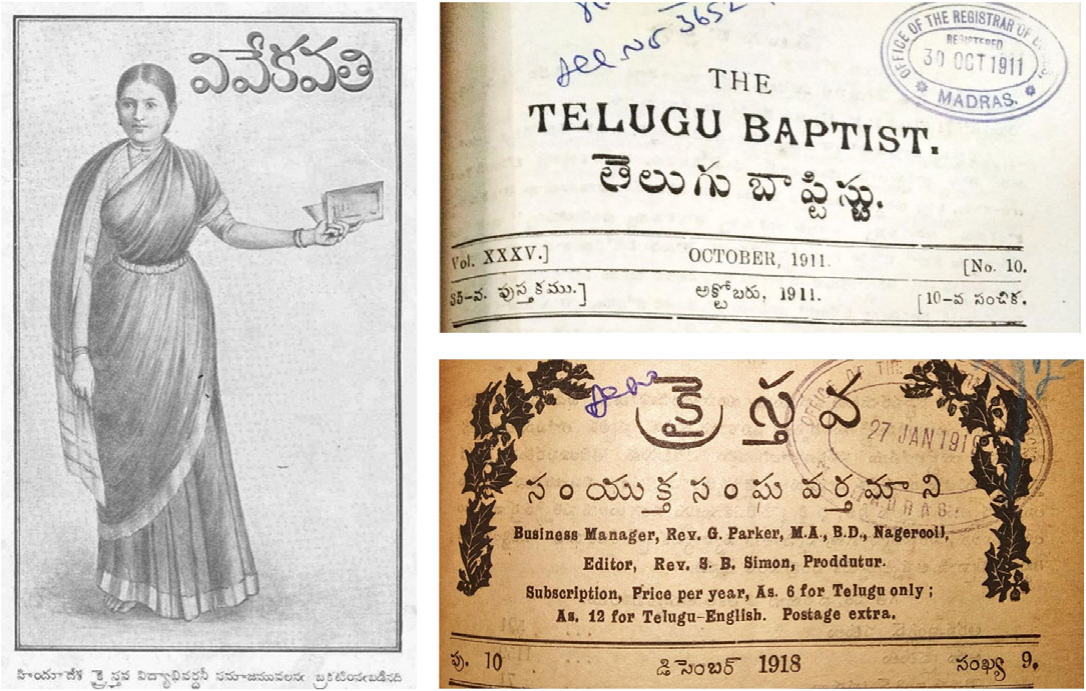
The three Telugu missionary journals, Vivekavathi, The Telugu Baptist and United Church Herald.

Methodologically, this article first brings the social history of medicine in conversation with Dalit Studies and Alcohol Studies. Secondly, it is attentive to the hybrid form of these journals, which packaged secular Enlightenment discourses in a web of Biblical language and medical truth claims.^6^ This hybrid form meant that medical temperance instruction was embedded in catechisms, hymns and allegories but at other times present in summaries of experiments. Finally, this article disaggregates terms like ‘universal’, ‘enslavement’, ‘addiction’ and ‘medical temperance’. The ‘universal’ is explored in medical Enlightenment and Dalit terms, in Biblical narratives of the clean and abstinent body, scientific assertions about the physiology of alcohol and its impact on digestion, liver and nervous system. The power of ‘the universal’ is disparately at work in both hegemonic and counter-caste voices. It also features in globalizing narratives – especially in the journals *UCH* and *The Telugu Baptist* – where alcoholism was shorn of caste associations and compared globally with drug and opium addiction. ‘Enslavement’ figures as a trope to free the body of its physiological addiction to toddy and arrack, liberate the Christian subject and free the drinker from fetters of caste stigma. In presenting ‘alcoholism’ as enslavement, the Telugu print sphere deployed a suite of overlapping terms and placed them in interwoven worlds of meaning.

## Situating *Vivekavathi* within the vernacular missionary world

The arrival of Anglican and Protestant missionaries across the nineteenth century meant that evangelical Christianity’s pathways into India engendered complex entanglements with empire. In that sense, it has been argued that there was no single ‘language’ or ‘framework’ that the Bible provided for ‘both interpreting and challenging imperial modernity’.^7^ The vernacular print sphere stood testament to this in terms of its hybrid genre that interwove medical and spiritual insights. Temperance expositions in these journals interspersed scriptural commentaries of the body as ‘the temple of God’ with scientific debates on whether alcohol was a stimulant or depressant, food or poison. Tamil, Telugu and Hindu missionary publications asserted that alcohol was a demon drink but also held toddy and beer to be lacking in nutritive content.^8^ They insisted on alcohol’s adverse impact on the physiology of the body, lactating mothers and child nutrition; its toxic chemistry; the unhygienic environments of toddy shops and the relationship between alcohol and heredity.

A notable illustration is a Tamil missionary publication termed *Kutumba Matuvilakku Castiram* [Domestic Temperance Science] authored by Y.G. Bonnell, meant for parents and teachers. Coming from a community of Nadars who were historically stigmatised for being toddy tappers, Bonnell strove subtly to fight against this taint of backwardness.^9^ As a teacher affiliated to the United Free Church of Scotland Mission and later headmaster of many schools, he was in a unique place to educate peers and students.^10^ This work executed a detailed foray into how alcohol changes brain chemistry, affects the processes through which blood clots occur, alters nerve function, obstructs gastric juice secretion and affects the kidney’s capacity to eject waste. Considered compositely, he wrote that alcohol wears down the immunity of the body to disease.^11^

In dispelling ‘myths’ that alcohol is nutritive food and in explaining why it is addictive, Bonnell resorted to temperance songs and parables that specifically castigate *kallu* or toddy and *sarayam* or arrack as dangerous. One such parable is the story of the swimmer who mistook a white bear in the middle of a river in spate to be a bundle of money. He jumps in to secure the bundle only to realise that it was a drowning creature desperate to cling to him to save its own life. He explained that the bear was alcohol that the alcoholic could not shake off and he warns the reader darkly against ‘brandy as the red bear’, ‘arrack as a black bear’ and ‘toddy as a white bear’.^12^

This co-production of medical and spiritual narrative occurred in both colonial Telugu and English missionary print. In English, the prolific volumes of *Abkari* (the mouthpiece of the Anglo-Indian Temperance Association), the *Indian Temperance Record* or the Indian publication of the Women’s Christian Temperance Union (WCTU) and Christian Literature Society (CLS) publications carried the voices of physicians alongside those of the medically informed missionary.^13^ However, the use of Christian narrative devices to impart medical pedagogy was more common to the Telugu print sphere.

*Vivekavathi* was an inter-denominational CLS monthly women’s periodical, which ran from 1909 to 1934. During its entire run, it endorsed the civilising quality of the British empire and American and British missions. It prided itself in carrying what the historian Mahaboob Basha termed the ‘white women’s burden of civilising the native woman’^14^ and endorsing reforms like widow remarriage and the abolition of child marriage. The editorial committee featured mostly white women and a couple of native Christians at any given time. As historian, Chakali Chandra Sekhar, who too worked on *Vivekavathi*, pointed out, white women missionaries from Protestant missions exercised tremendous agency in shaping this journal.^15^ That said, the contributors were significantly though not exclusively Indian Christian converts and many of them women. In choosing a term like *Vivekavathi* or ‘the wise woman’, the editors positioned themselves as a secular alternative to other Christian publications, such as *UCH* and *The Telugu Baptist.*

Basha wrote that the monthly periodical had thirty subscribers to begin with, a number that went up to 1 400 by October 1913.^16^ It had thirty-two pages in the first issue. Its annual subscription rate was twelve annas (or 1/16 of a rupee) by the end of the first year of October 1910. To cite one of *Vivekavathi*’s editors, Elizabeth McCauley, subscribers and readers included converted Christians, merchants, women teachers, Brahmin widows and ‘progressive Hindus’.^17^ McCauley implies that Dalit readers of *Vivekavathi* were a large subset of converted Christians. She infers Telugu Dalit readership of the journal from the high literacy rate of converted Christians from the Madras Presidency region in the 1911 census, on the one hand, and their contribution to the journal, on the other. She adds in no unmistakable terms that converted Christians, for the most part, were ‘formerly the despised and oppressed outcastes’.^18^

Given that this was a women’s journal, some articles were targeted at them, as for instance, the offerings of domestic temperance science in the column termed *Sundara Gruhamulu* or ‘The House Beautiful.’ Many others were more broadly categorised and heavily medicalised, these series included *Arogya Vishayamulu*, or ‘Health Matters’, *Madyanishedhamu* or ‘Prohibition’/‘Temperance’ and *Patrika Sampadakurali Vyasamulu*/*Sampadakulu* or ‘editorials’. These articles were particularly invested in defining the characteristics of the alcoholic.

Between 1909 and 1926, roughly sixty articles on subjects such as alcohol, inebriation, intemperance and domestic temperance science were featured in *Vivekavathi.* Articles on temperance added up to a total of five in the inaugural year of the journal, i.e. 1909, and were most plentiful between 1915 and 1920, totalling up to thirty-five. The monthly issue of October 1919 alone saw seven articles published on the subject. While it is almost impossible to state exactly why this period saw a jump in the number of articles on temperance, it could have converged with the lead up to the anti-liquor agitation in the Madras Presidency. While the latter picked up momentum in mid-1921, heated discussion of the rise in liquor prices and increased alcoholic consumption among labourers took place well before this, in the year 1917.^19^ However, *Vivekavathi* did not present temperance debates in a nationalist vein unlike a popular, contemporary Telugu newspaper, *Andhra Patrika*, choosing rather to approach them as weighty scientific and spiritual matters.

Even though it was not a mouthpiece of WCTU, *Vivekavathi* embraced the ‘internationalism’, ‘American values’ and the ‘Anglo-American roots’ of this organization.^20^ The journal carried several articles by British and American medical missionaries and WCTU figures like Maud Allen and Margaret Denning. It used the genre of the temperance hymn, very common to WCTUs and the Band of Hope’s temperance campaigns.^21^ Authors also cited renowned British and American medical figures like Victor Horsley, Mary Sturge, Leonard Rogers, Frederick Treves and Henry Beaumont. Their medical findings about the harmful chemistry of alcohol and its role in altering the physiology of the human body were presented as universal truth claims.

These medical forays interspersed with international examples of scientific experiments were in tune with the journal’s intention to ‘open up perspectives of things happening around the world, with the blessings of God and how they came into being’.^22^ While *Vivekavathi* served universal narratives on a Biblical platter, it did so within a deep-seated regional framework. The cultural modernity of temperance is to be situated not so much in *Vivekavathi*’s turn to Western medicine and science as much as in its (mis)translations, interpretations and regional formulations. Medico-scientific theories became a vehicle for expressing hegemonic and stigmatising caste-mediated propaganda that associated toddy and arrack with marginalised castes, and Dalits in particular. Counter-caste narratives saw the same British and American medical theories as facilitating something radically different, a temperance campaign for Dalit and marginalised caste self-respect. The feminist historian, Shailaja Paik refers to the ‘micro-transformation’ of ‘upper caste’ values for a radical politics of anti-caste self-assertion.^23^ This article builds on Paik’s arguments to demonstrate that universal medical theories, espoused by upper castes, were also the site of counter-caste self-making.

Rather than convey ‘enslavement’ to drink in purely medical terms or overarching terms as ‘a disease of the will’,^24^ hegemonic counter-caste voices mingled the social, spiritual and medical. The enslavement of the alcoholic was explained as being oblivious to the medical hazards of drinking for children and women, the toxicity of brandy and some peculiarly minute descriptions of toddy and arrack. These drinks were described as contributing to secondary medical conditions like *ajeernamu* [indigestion], *kshayarogamu* [tuberculosis], *vaatarogamu* [gout], *moorkharogamu* [epilepsy] and *rompa* [cold and chills]. Counter-caste voices called for respecting one’s body as the temple of God, upholding the Nazarite vow while making a case for the dismantlement of especially toddy and arrack’s strangle-hold over oppressed lives in terms that were medical, social and Biblical.^25^

The last section of this article briefly examines two other Telugu missionary journals, *Kraistava Samyukta Sangha Vartamani* or *UCH* and *The Telugu Baptist.* Not much is known about these other two journals except that *UCH* was published in two separate editions (Telugu and Telugu-English). Its publisher was not CLS Press but Minerva Press.^26^ *UCH* occasionally re-published articles from *Vivekavathi*, and both journals emulated *Vivekavathi*’s style of using bilingual titles. While they too deployed medical narrative to denounce alcohol, they were not inclined to comment on local power structures and the caste dynamics of consuming toddy and arrack. They thus held out less potential for counter-caste narratives.

## Toddy and arrack in English and Telugu colonial print culture

The stigma underlying *Vivekavathi*’s subtle and not so subtle aversions to toddy and arrack must be read within a longer socio-medical history of caste in the vernacular and English print sphere. Far from being innocuous, these graphic associations were laden with the historicity of caste churnings around alcohol in both the Tamil- and Telugu-speaking parts of the Madras Presidency and the Hyderabad state during the early twentieth century.

C. Rajagopalachari or Rajaji, as he was popularly known, was at the helm of the Tamil Nadu Congress Committee–driven nationalist agitation against alcohol in Madras Presidency. He sought to mobilise public opinion through his unremitting campaign against toddy renters and arrack ‘vends’ (or vending spaces) for the nationalist daily, *The Hindu.* He assiduously published excerpts of his conversations with scientists researching nutrition at the Pasteur Institute, Coonnoor. He reported that toddy was less nutritious than buttermilk because it did not contain as much protein and its vitamin content was nothing out of the ordinary.^27^ He also reinforced caste stigma when he raged at Hindu temples for leasing out coconut trees for toddy-tapping. He wrote about one such temple, the Triplicane temple in Madras city: ‘This ancient temple was intended for a civilised form of worship; but you have converted this into something like those temples where goats and fowls are slaughtered’.^28^

Detailed ethnographies of ‘castes and tribes of South India’ figured in the deeply Orientalised and racialised writings of anthropologists like Edgar Thurston. Like Rajaji, Thurston clubbed together familiarity with toddy and animal sacrifice. In addition, he coupled drinking toddy and arrack with demon worship and flesh-eating practices among castes and tribes whom he regarded as backward.^29^ Missionary publications in English produced a rich corpus of richly medicalised writing condemning toddy and arrack as particular to backward castes.^30^

But these associations were also common in the Telugu public sphere across nationalist, imperial and niche health journals. A Telugu weekly titled *Andhra Harijan* made associations between toddy and arrack and marginalised castes in explicit terms when authors referred not only to toddy trees as intrinsically unhealthy but also their byproducts as also evil.^31^ While this establishes a general pattern to make caste-laden associations between toddy and arrack, it is possible to go one step further and discuss the more overarching trend to anchor health norms to caste within Telugu journals and newspapers. A monthly Telugu health journal called *Arogya Prakashika*, which was, inter alia, invested in propounding medical advice surrounding plague and tuberculosis, featured several articles on alcohol. These articles were peppered with ample references to how alcohol ravages the nervous and digestive system, solidifies food instead of breaking it down, increases cholesterol in the body, hastens the heartbeat and lowers immunity to plague. Toddy and arrack featured in temperance narratives that equated them with castes that were regarded backward. An article gestures to the dangerous habits of toddy and arrack consumption among rickshaw pullers and midwives before they go to work.^32^ Another article discusses how arrack destroys the nervous system and causes mental disorder while simultaneously citing Hindu texts that warn that it is ‘more sinful to consume alcohol than kill a cow’.^33^ This journal, much like *Vivekavathi*, featured overlapping scriptural and medical narratives, caste narratives and citations of British and American authors. But the scriptural narratives of *Arogya Prakashika* were drawn from Hindu texts rather than the Biblical cannon.

## Reading caste in *Vivekavathi*’s medical temperance campaigns

The medical associations between toddy, arrack and caste are stark in *Arogya Prakashika* but they are mostly veiled in *Vivekavathi.* There is, however, a more distinctive imperial function that such articles in *Vivekavathi* carry out. While *Vivekavathi*, as Basha points out, sought to raise the health standards of natives, especially women, they also executed a subtle caste-driven mission to educate marginalised castes and tribes perceived to be lacking in basic medical precepts.^34^

This becomes manifest in an editorial that describes an initiative to secure temperance pledges – termed *vagdattamu* in Telugu – from the parents of children in ‘Mala villages’, who were seen to be shockingly familiar with toddy and unfamiliar with nutrition.^35^ The article goes on to say,

We have been working with schools for some time now and in this connection, we visited a big Mala village. When we asked the question, who among you drinks *kallu*, forty students including the very small children raised their hands.^36^

The same editorial cautions readers in a dual sense against parents offering drinks to their children and the consequences of early alcoholism among adults before and after marriage for children. It cites medical data about child and adult mortality both in the Rayalaseema region in the Madras Presidency and England. The tendency to cite drinking statistics with relation to life expectancy, maternal health and hereditary disease was very common in British and American medical and missionary temperance publications.^37^ The *Vivekavathi* editorial claimed that children of drinking families in England were likely to develop conditions of muteness, epilepsy and tuberculosis. In Rayalaseema, a southern region of the Hyderabad state, parents with no pre-existing drinking habits lose thirteen children out of one hundred on an average every year, while parents with mild drinking habits lose twenty-three out of one hundred and those with extreme drinking habits lose thirty-two out of one hundred .^38^

The empirical descriptions of the drunkard in the Telugu context as ‘Mala’ and drunkards as ‘Mala villages’ stand out in this editorial. The warning about the potential loss of nutrients in breastmilk among those familiar with *kallu* and *sara* [arrack] is coated with the caste conviction that it is Mala mothers who are particularly culpable of alcoholism. The editorial subtly warns readers, in pedagogical terms, against emulating them.

Another article distinguishes drinking brandy and whisky in clubs, parties and lavish dinners with *kallu* and *sara* consumed by ‘coolies’, ‘fishermen’, ‘cotton fields’ and ‘factory workers’ as well as those working for the *Dora.* This is a caste-laden Telugu term translated as powerful landlord or lord – over the land, the fields or a part of the village – where Dalits and marginalised castes had to render slave labour and other services in a system of caste-based slavery called *vettichakiri.* ^39^ In *vettichakiri*, Dalits had to render caste-based (slave) farm labour while other marginalised castes had to provide free caste services in their capacity as barbers, washermen, toddy tappers and potters. Those marginalised castes who tapped toddy had to set aside trees exclusively meant for landlords and their families.

Writing in a deeply casteist register, the *Vivekavathi* author decries these elite and by extension upper caste drinkers for succumbing to peer pressure and marginalised castes for drinking out of bodily exhaustion and ignorance of hygiene and health norms. This is manifest in descriptions of how toddy and arrack are usually paired with *manchi vasana gottu padu kooralu* or ‘curries reeking of rotting vegetables’ served near the liquor shop.^40^

The same article argues that alcohol physiologically enslaves: the alcohol-habituated body is one in which disease spreads rapidly and effortlessly. When a fatigued body encounters alcohol, it causes the activity of the *naramulu* or the nervous system to slow down, with the result that the person feels pleasure and believes that alcohol is good for him/her. This slowed-down physiological activity caused especially cotton field workers, factory workers and coolies to become drunkards and worship *Saramma* and *Kalludevi* – made-up disparaging names for ‘lower caste’ goddesses dedicated to toddy and arrack and behave like foxes, tigers and pigs.^41^ These deeply stigmatising caste associations were packaged in universal observations pertaining to hygiene, the body’s physiology and the altered chemistry of the mind. What is however unmistakable is the coupling of classes – that are usually from marginalised castes – such as coolies, factory and field workers with bodies that were slowed down and enslaved by toddy and arrack.

*Vivekavathi* also made lengthy forays into what they presented as cutting-edge medical science, replete with irrefutable findings on the physiology and physiological effects of alcohol. Several articles delve into the scientific virtues of temperance on the strength of experiments while foregrounding the vile character of toddy and arrack. For example, an unnamed author alludes to experiments conducted by a ‘Dr Monroe (sic)’ from Hull. This experiment focuses on what occurs to beef, when it is cut into portions and when it is immersed in water, toddy and arrack, each of which was mixed with gastric juice in separate containers and maintained at 100°, to simulate a natural stomach situation. The Telugu article summarises the findings thus: when immersed in water, the digestion of the beef was well under way in four hours, that it broke down into chunks after eight hours and that the beef dissolved into the gastric juices after ten hours. His findings with toddy showed that the beef turned black and gave rise to black foam after four hours, that it broke down after eight hours and that it did not dissolve even after ten hours. With arrack, his findings were that the beef ‘remained the same’ after four hours and eight hours and that it became *gatti* or ‘rigid’ after ten hours.^42^

There were some key differences between this Telugu version and the original Dr Henry Munroe text, titled ‘The Physiological Action of Alcohol’, and another archival text by an American physician, Dr William Hargreaves. These were both temperance texts that were not influential in the British and American medical and missionary landscape. This could be because these two temperance figures summarised and consolidated important experiments in chemistry and physiology but did not themselves pioneer pathbreaking research. These texts were, however, regarded as good reviews of temperance literature along the lines of the chemical composition of alcohol, alcohol as a narcotic, its impact on digestion, its role in diseases affecting the different organs and diseases affecting the mind and hereditary disease.

The Hargreaves version contained a detailed exposition of the *Vivekavathi* experiment with a table very closely resembling that used in the Telugu version.^43^ Far from experimenting on *kallu* and *sara* as described in the Telugu version, they featured experiments with the more generic ‘alcohol’ and ‘pale ale’.^44^ In the Munroe version, the experiment involved a mixture of bread, meat and gastric juice, to which ‘a glass of pale ale or a quantity of alcohol’ was added.^45^ He instructs the pouring of this mixture into a phial, which could be immersed in a sand-bath maintained at a low heat of 98º. He recommends the occasional brisk shaking of the contents in tune with the stomach’s movements. He states that ‘at the end of seven or eight hours, or even some days, the food is scarcely acted upon at all’.^46^

In Hargreaves’ text, Dr Munroe mixed finely minced meat with gastric juice from the stomach of a calf with water, alcohol and pale ale and kept the bottles at a temperature of 100°. His observations of what happened to the beef are similar in both the Hargreaves and the Telugu *Vivekavathi* texts with varying details of the different hours of observation. The descriptions of Dr Munroe’s inferences – namely that alcoholics destroy the solvent power of gastric juice and prevent digestion – are similar in all three versions.

**Figure 4:**
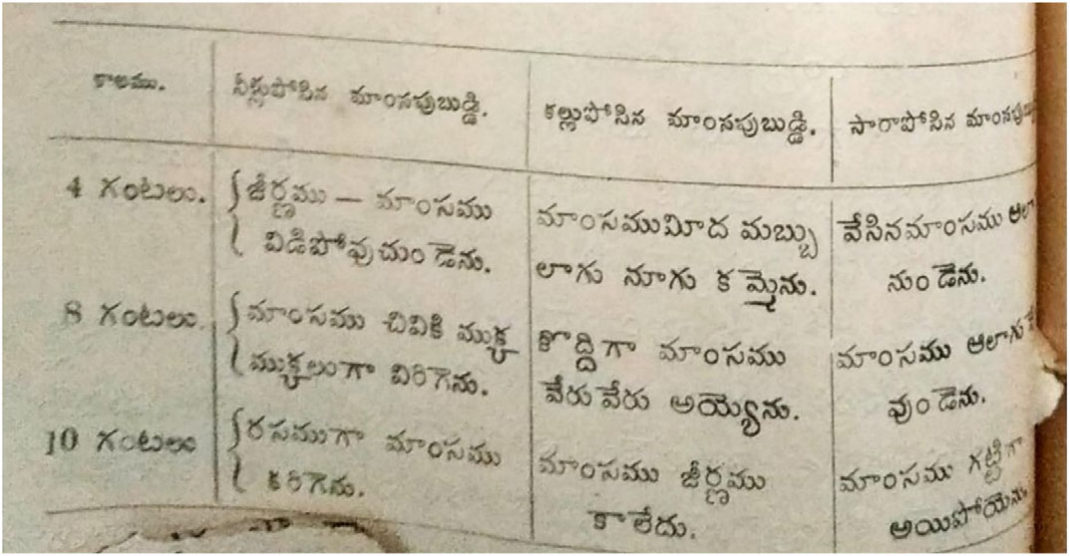
Table describing experiment with toddy and arrack in Vivekavathi; Source: AP State Archives.

Assuming that the *Vivekavathi* author used the Hargreaves version – which was almost certainly the case given the similarity of tables (Figure 4) – the substituting of alcohol and pale ale with toddy and arrack is unmistakable. One can argue that the *Vivekavathi* author used toddy and arrack to render the article more context-friendly and took creative liberties; Munroe did say in his original text that any alcohol can be used. However, there can be no escaping the stark motif of toddy and arrack as particularly needing to be shunned across *Vivekavathi*’s socio-medical landscape.

In replacing pale ale and alcohol with toddy and arrack, *Vivekavathi* may have conflated Munroe’s findings about pale ale set in a British context with the common views of physicians and tropical disease epidemiologists like Norman Chevers and Leonard Rogers. Historian Erica Wald writes that Chevers excoriated toddy and arrack in the colonial context as a potent ‘cocktail of drugs and hallucinogens’.^47^ These drinks were seen to be all the more dubious owing to the local toxins they contained. Where claret and beer were seen to be befitting of higher European classes in a tropical environment, toddy and arrack were demonstrated to be routinely consumed by ‘lower-orders’ among British soldiers, sailors and Indians and that too in deeply unhygienic surroundings marked by open sewers.^48^

Another *Vivekavathi* author pays homage to two famous medical temperance figures, Victor Horsley and Mary Sturge, by deploying the same title of the text they authored, ‘Alcohol and the Human Body’. This article cited the statistical medical figures in the original work, that in seven major hospitals of London, the expenses on alcohol were 12 000 pounds or 120 000 rupees in 1852 and those on milk were 3 000 pounds or 45 000 rupees. Over the next few years, London’s hospitals spent less than 3 000 pounds on alcohol while increasing their milk expenditure to 8 000 pounds.^49^ The author also asked the same question as did Horsley and Sturge, i.e. ‘what is alcohol?’ The answers were exactly similar as well, with both texts explaining fermentation to be a process where the yeast plant acts on sugars such that it splits them into alcohol and carbon dioxide or carbonic acid gas. However, the Telugu author goes from here to illustrate fermentation saliently through different types of toddy sourced from the palmyra, date or coconut trees.^50^ This stood in stark contrast to Horsley and Sturge, who considered the medical impact of a panoply of drinks ranging from beers and wines to distilled liquors such as whisky, gin and schnapps on digestive processes.^51^ The caste-laden condemnations of toddy and arrack in *Vivekavathi* were thus marked in a universal language of medical discovery.

## Counter-caste voices: The power of the ‘universal’

No narrative other than a strictly temperance-driven one was possible considering *Vivekavathi*’s editorial stance. Given, however, that it was a field of universal claims and assertions, it was one that enabled counter-caste voices to express themselves. This article’s usage of ‘counter-caste’ owes much to literary historian and Dalit Studies scholar, Satyanarayana’s deployment of ‘counter-publics’ or oppositional, anti-caste voices who broke ‘invisible’ barriers of articulating their identities in secular and liberal spaces.^52^ These voices sought not so much to preserve caste stigmas against alcohol but to ensure that those from marginalised backgrounds are freed from them. In so doing, they too relied on universal Biblical and medical narratives.

The term ‘universal’ has a place both within Dalit Studies and critiques of ‘Medical Enlightenment’. Scholarship around the latter demonstrates that the universal empiricist, rationalist and naturalist interventions of Enlightenment were to the effect of presenting medical knowledge as ‘certitudes’ and proven truths rather than mere ‘conjecture’.^53^ But as Maurice Finochiaro argues, the ‘empirical’ was a tool in the arsenal both of those like Galileo and Copernicus and those who opposed him.^54^ In the early twentieth century – the same timeframe of this article – German brewers and producers executed their own stakes in the ‘Medical Enlightenment’. Michael Hau writes that they mobilized medical arguments that alcohol was harmless and that it boosted immunity to tuberculosis, nervous disease and related morbidities.^55^

Medical Enlightenment scholarship however did not bring the universal in conversation with inequality whereas Dalit Studies addressed genealogies of the universal in pursuit of self-empowerment. Historian and Dalit Studies scholar Sanal Mohan writes that CMS missionaries synthesised Enlightenment ideals with Biblical concepts of salvation in addressing caste-based slavery in Kerala. They deployed terms like ‘rights-bearing individual’ and ‘suffering slave’ while failing to discern slavery within the world of agrarian caste-based exploitation.^56^ Converted Dalit slaves, however, were able to appropriate ‘universals’ and embrace missionary pedagogic habits of ‘new sartorial and bodily practices, acquisition of language, literacy and education’ to regain their humanity.^57^

A historian and Dalit Studies scholar working on the Telugu print spheres, Chinnaiah Jangam, brings a different critique by arguing against the singular acceptance of the premises of contempt for ‘universal principles of Enlightenment’ underlying Postcolonial Studies.^58^ If this discipline rejected such principles owing to their Eurocentric heritage, Jangam shows that Dalits experienced the colonial state as well as missionary presence in Telugu public sphere differently. Given the state of agrarian slavery and violent segregation that Dalits lived in, it was the sustained administrative efforts of the colonial state and missionaries that yielded results of accessible public education for Adi-Andhras. He concludes that liberalism, when channelled to fight ‘caste-based violation’, created avenues of ‘ideological power’ to Dalits.^59^

Counter-caste voices in *Vivekavathi* were able to appropriate universals, such as ‘alcohol as a narcotic’, alcohol as ‘causing bodily heat’ and ‘destroying the digestive tract’, packaged in parables and catechisms. These universals were very enabling in calling out the enslaving stigmas that toddy and arrack subjected marginalised communities to. But they also turned WCTU songs into temperance songs that attacked the very local manifestations of debt, landlordism, caste stigma, inequality and loss of self-respect.

*Vivekavathi* authors like Hariyatamma, Nagulaiah, M. Augustine Narasimhulaiah, Pendurti Joseph and O. David were driven by Dalit sensibilities in that they spoke out against Brahminical ritualism, dehumanising upper caste scriptures and caste hierarchies on spiritual grounds. For example, an author presented a dialogue between ‘a Dhoby’ (regarded the ‘washerman’ and ‘low caste’) and ‘a Brahmin’, which entails the former challenging the latter’s backward beliefs about a sacred river being capable of wiping all Sin.^60^ Counter-caste voices also marked out the alcoholic’s ‘enslavement’ to drink and local caste hierarchies of toddy and arrack even as they strove to disseminate medical knowledge about temperance.

They narrated analogies and stories that revealed subtle ways in which they raised questions, chiefly whether medical knowledge was the monopoly of upper castes and why scientific discoveries about alcohol elude Dalit and marginalised subjects. Nagulaiah, a *Vivekavathi* author, wrote that temperance contributed to healthy bodily practice and explicitly indexed *sara* as bringing about the loss of dignity and self-respect. Writing in deeply allegorical terms, where he conjured the self-harm that *sara* can cause, he narrated the story of the *Yogi* or Saint Samuel – presumably the same Samuel who features in the First Book of Samuel – who brought about the reform of an abusive drunkard husband and father. While the allegory was placed firmly in a Telugu context and featured plot elements removed from the First Book of Samuel, certain imagery from this part of the Old Testament cannot be missed.

Upon witnessing a woman suffer on account of her drunk husband, Samuel intervened to pay off the debt the latter incurs when he drinks *sara* without paying the *sara* seller. The husband subsequently had a dream involving four mice – or *chaturmooshikamulu* – where the first one was fat and aggressive; the second weak, malnourished and poor; the third in a dying condition and the fourth blind. Samuel explained to the young man that the first mouse represented the *sara* seller, the second represented his enervated and helpless wife, the third his children and the fourth blind one none other than the drunk man.^61^ While Nagulaiah did not deploy medical wisdom in this temperance allegory, the imagery of the mice representing the fat *sara* seller, the dying children of the drunkard and the malnourished wife are hard to miss. Equally significantly, the point of this story was to explain how alcohol caused disease and not only loss of wealth, intelligence, health and life but also self-respect.

Medical themes are present in this article when considered in a hermeneutic sense. In the King James Bible, the First Book of Samuel 6: 4 and 6: 5 gestures to mice as signifying ‘plague’, disease and poverty – made explicit in ‘mice that mar the land’. Nagulaiah’s allegory brought these Biblical meanings to bear on a Telugu landscape, where *sara* dealers held their customers in thrall, commonly those from marginalised castes. Akin to what the historian R.S. Sugirtharajah indicates, non-Brahmin castes and converted Christians have appropriated the Old Testament for ‘hermeneutic interpretation’, where it resonated with their ambitions to fight caste.^62^ Even though Nagulaiah did not use the term ‘caste’ itself nor does he cite a caste name, it is unmistakable that in his allegory, alcoholism is enslaving in medical terms of disease and impending death and socio-economic terms of debt, both themes that recur in several Dalit and marginalised accounts.

An attentiveness to how alcohol enslaves and eats away at one’s self-respect, even as it enervates the body and those around the drunkard, stand out here. This article in *Vivekavathi* cannot be seen in isolation; these interpretations of *sara* as an ‘unclean’^63^ beverage that enslaves Dalits find resounding echo in contemporaneous Telugu Dalit writings. Historians and Dalit Studies scholars, Chinnaiah Jangam and Sambaiah Gundimeda, provide detailed references to leading early-twentieth-century Adi-Andhra or Telugu-speaking Dalit intellectuals and reformer-activists. Gundimeda writes that the term Adi-Andhra was critical to the self-naming practices of Telugu-speaking Dalits in the Madras Presidency – it meant those who were the original inhabitants of the Andhra region.^64^

Prominent Adi-Andhra activists, such as Bhagya Reddy Varma, Jala Mangamma, Jala Rangaswamy, Gangaiah Rayudu, Arige Ramaswamy and Kusuma Dharmanna, came up with minute socio-medical strictures against alcoholism as reinforcing spirals of debt, poverty and disease in their communities.^65^ Jangam indicates that these leader-intellectuals signalled the political charge of collective self-transformation that temperance held out to Adi-Andhras’ assertions of self.^66^ He argues that their many intellectual and political legacies of publishing journals, initiating social reform, moulding the political arena and public opinion on matters including temperance underscored their role in shaping nationalist modernity.

The scientific basis of temperance was sharply conspicuous in contemporary Adi-Andhra writings. Gorantla Rangaiah, an author writing for The *Bhagyanagar Patrika*, a Dalit fortnightly newspaper, wrote in a medical voice against narcotic addiction. He expresses alarm at the rising trend in Hyderabad schools of using *mattu vastuvulu* or narcotic substances under peer pressure. He wrote that children could incur serious and early risks to their bodily and mental health when they ‘rolled cigars, did snuff and got hold of bidis and cigarettes’. ^67^ All this served as a precursor to the most dangerous health hazard of drinking alcohol. But perhaps most notable of Adi-Andhra temperance advocates was the medical voice of Kusuma Dharmanna and his impressively detailed medical tract called *Madyapana Nishedhamu*, the same title that O. David had earlier used for one of his temperance songs, even if these may be unrelated events.

Kusuma Dharmanna’s work, *Madyapana Nishedhamu* [Temperance], draws on the body as ‘the house beautiful’ analogy in that just as the house has its pillars, beams and walls, bones and muscles perform similar functions and the human the proud owner of the house.^68^ He shows how the well-oiled and well-functioning body is ruined when alcohol is introduced to it and how it causes havoc especially to respiratory, digestive, brain, muscle and other vital aspects. He discusses the nature of the human body, the water content in it and that far from being a food, alcohol is a poison that causes imbalance. He narrates the diseases that ensue from alcohol and warns against administering toddy to children.

**Figure 5:**
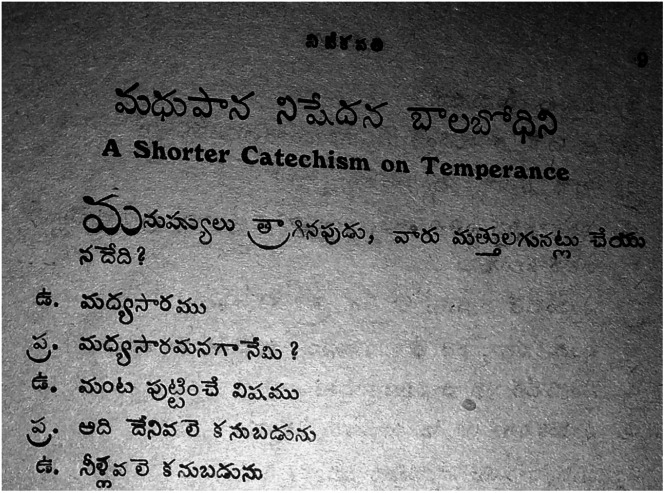
A temperance catechism authored by Pendurti Joseph explaining fermentation and likening alcohol to a corpse preservative.

Written roughly around the same timeframe, a few articles in *Vivekavathi* resemble Kusuma Dharmanna’s medical pamphlet, in their tone, analogies and citation of scientific discoveries. Notable among these, although written prior to Dharmanna’s tract, is Pendurti Joseph’s medical temperance catechism (Figure 5).^69^ Both authors wrote in an entirely medical register to describe alcohol as an unnatural poison and outlined what fermentation does to alcohol. They described alcohol as a corpse preservative and cited experiments in which fish and snakes die when they are dropped in water with even one per cent alcoholic content.

Both Pendurti Joseph and Kusuma Dharmanna invoke the power of universal medical knowledge implicit in the latest scientific findings of the day on the toxicity, nutritional outcomes and bodily processes informing alcohol consumption. Their analogies demonstrated knowledge of the experiments and summaries in the writings of physicians such as Benjamin Ward Richardson, Victor Horsley and Mary Sturge. Horsley and Sturge’s famous work, *Alcohol and the Human Body* finds mention in quite a few medical temperance articles in *Vivekavathi*, especially on ‘the medusa’ or the fresh-water jelly fish.^70^

It is this performative register of bringing the latest medical findings to bear on the stigmas of Dalit bodies and livelihoods that Dharmanna definitely deployed and Joseph may have also undertaken. G. Subramanyam, in his introduction to Dharmanna’s *Madyapana Nishedhamu* – which won the best prize in a competition set by a regional temperance association – wrote that even if this was a medical text, the author was all too aware that alcoholism was not so much a personal addiction as a social disease that weighed Dalits down like a *rugmata* [sickness].^71^ What is no coincidence is also that Dharmanna was an Ayurvedic doctor by profession who made extensive socio-literary interventions against untouchability, critiques of Hinduism and caste landlordism.^72^

While not enough is known about Pendurti Joseph, other similar voices in *Vivekavathi* refrained from stigmatising Adi-Andhras or Dalits and were embedded in a critique of caste hierarchies. They could have emerged from similar considerations as Dharmanna’s, namely that temperance was an article of Dalit self-respect. These inflections set these authors apart from stigmatising voices whose disgust towards toddy and arrack occupied a scriptural-moral plane that upheld upper caste values of temperance.

**Figure 6:**
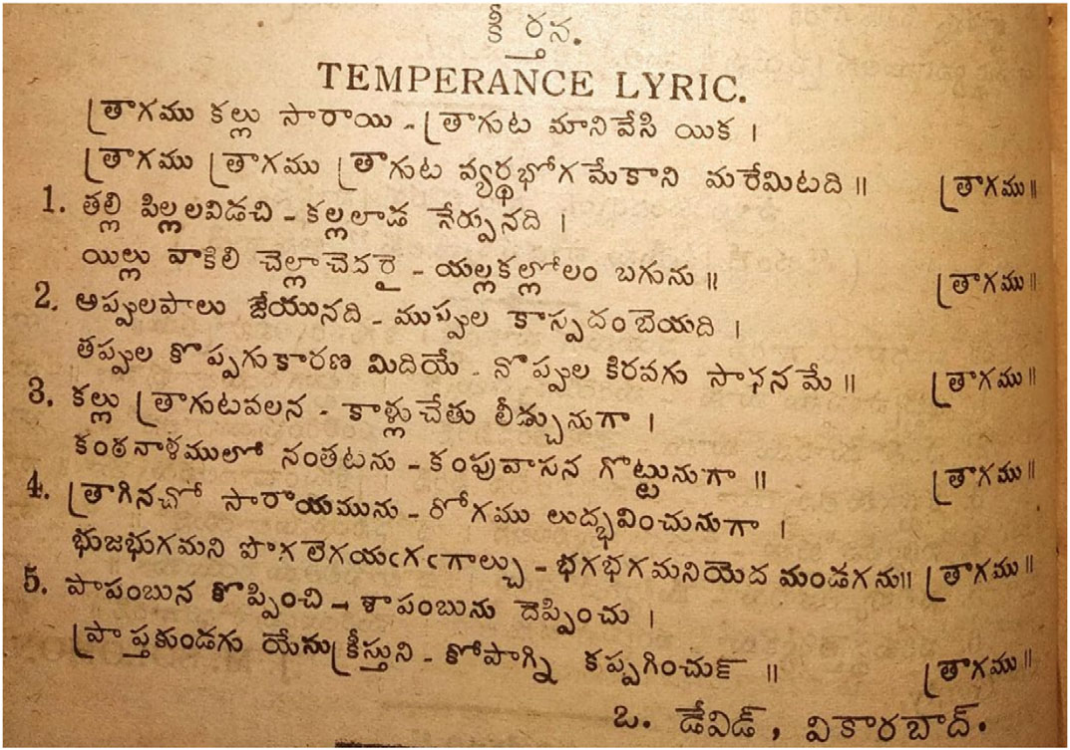
A temperance hymn in Vivekavathi that melds social and medical conceptions of enslavement.

Another voice that stands out in *Vivekavathi* is that of O. David, who authored many ‘temperance songs’ and ‘temperance lyrics.’ In one article, he implicitly condemned alcohol by laying out commandments for a healthy body. He wrote that consumption patients particularly needed fresh air, clean food, well-rested body, a reviving environment and a temperament of dwelling on positive things.^73^ On another instance, he suggested that temperance was critical because it ‘enslaves’ the alcoholic to debt (see original in Figure 6):

*Toddy enslaves you to debt*
It is the origin of dangers
It is the biggest cause of mistakes
It is the instrument of pain
On account of drinking toddy,
Your hands and feet give up on you
Your throat and passages
Are afflicted by a stench
When you drink arrack
You are causing diseases
When you smoke cigarettes
Your chest and heart burn (emphasis mine)^74^

In O. David’s verse, enslaving debt and social ruin coalesced with a sensory assessment of disease. Alcoholism referred to a social state that was mirrored in the disease-prone and collapsing state of the body marked by chest-burn and a reeking throat. Neither O. David, Nagulaiah nor any other counter-caste voice in *Vivekavathi* challenges toddy and arrack as stigmatised objects, in medical or other terms. However, they do underline the trauma caused by social stigma to the alcoholic.

**Figure 7:**
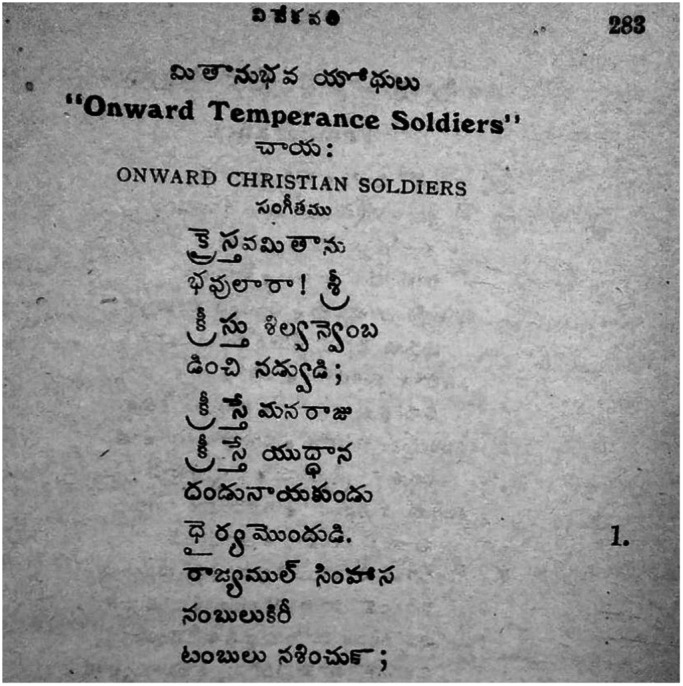
The Vivekavathi temperance hymns draw on WCTU temperance songs, but feature local interpretations of toddy and arrack.

The form and content of these temperance songs pointed to a hybrid genre. On the one hand, they were inspired by the WCTU temperance hymns and featured the same titles such as ‘Matanubhava Yodhulu’ or ‘Onward Christian Soldiers’ (Figure 7). On the other, they were distinctive in that they contained themes of ‘bondage’ to alcohol.^75^ While the WCTU version conjured a marching army united in their path of the Cross and acceptance of Jesus as leader, the Telugu version featured, in addition, a passionate appeal to forgo debt and the stench of local beverages.^76^ If the WCTU hymns featured classical Western musical notations, O. David’s hymns conformed to upper caste musical tastes in that these songs took the form of a *keertana* (classical Carnatic music form) set to *taalam* and *raagam.* ^77^ However, the same song was unmistakably local in its appeal to forgo toddy and arrack.

The local here was embedded in anti-caste movements. Even if the form of the song was upper caste, the genre of the temperance hymn had Dalit roots. Temperance hymns were popularised by Adi-Andhra leaders like Jala Rangaswamy and Jala Mangamma.^78^ Chinnaiah Jangam states that writings by Gandhian Dalit leaders like Rangaswamy denouncing alcohol were definitely shaped by upper caste ‘strictures imposed by reformist Hindu parameters on the Dalit worldview’.^79^ However, he argues against dismissing these writings for they recast history to enable a Dalit nationalist modernity. These complex social formulations were evident in *Vivekavathi* too.

The same O. David decried *kallu* and *sara* by implicating them within a system of sexual slavery forced by Hindu society on Dalit communities like ‘Malas’ and ‘Madigas’.^80^ In such a system, girls were forced by upper caste Hindus to become ‘joginis’, a term used usually to refer to Dalit women who were coerced into ritual prostitution. Requiring joginis to drink toddy and arrack was all too common, he writes. His angst against sexual slavery was reflected in contemporary Dalit women’s writings.^81^ In the same article, he expresses consternation against the increasing enslavement of Dalits to these drinks owing to the ubiquitous natural feature of the *eeta chettu* or the date palm tree in every village in the princely state of Hyderabad. He implies that Dalit parents wrongly perceived date toddy to have nourishing qualities since they fed even their infants with this.^82^ Reading O. David’s various articles together, it can be argued that he simultaneously rejected narcotic substances because they compromised every scientific principle of clean food, caused secondary diseases like tuberculosis and entrapped Dalits further in caste-based slavery.

## Historiographies of medical temperance, alcoholism and enslavement

This article relies on scholarship gesturing towards the complementarity of missionary and medical languages in driving dominant discourses of ‘alcoholism’. In this regard, historians have globally engaged with medical temperance within overlapping frameworks of vice, epidemic disease, social purity, hygiene and eugenics. The essays published in the edited volume *Global Anti-Vice Activism* see alcohol as a site of medicine’s civilizational phobias and missionary anxieties manifest in panics around ‘racial hygiene’, ‘racial poison’ and ‘venereal disease’.^83^ Nikolay Kamenov wrote that scientific discourses on ‘alcoholism’ in interwar Bulgaria and the Balkans region recast the WCTU missionary language of ‘social and moral hygiene’.^84^ In his work, temperance visual culture took the form of posters, magic lanterns and pamphlets to warn against the addictive aspects of alcoholism. Roy Porter traced the disease concept of alcoholism to Georgian England and Christian evangelical narratives.^85^

In this article, however, missionary publications did not merely pave the way for a more medicalised language to emerge. They constantly cited Anglo-American medical narratives and resorted to heavily medicalised debates on digestion, toxicity, nutrition and addiction and featured minute descriptions of how alcohol affected the muscles, liver, brain and heart. But even in 1930, Telugu missionary journals did not use the medicalised terms, *vyasanamu* or addiction and *vyasanaparudu* or addict, but rather gestured towards ‘alcoholism’ through terms like *tragudu* or drunkenness*, tragubothu* or drunkard, *nisha* or intoxication*, mattu* or narcotic state and *lobaduta* or enslavement.

Drink historians have used the term ‘slavery’ in depicting subtle processes of the medicalization of alcoholism in Britain and Europe. Mariana Valverde and James Nicholls furnished commentaries on the institutional vigour, legislative power and disciplinary force undergirding medical theses about alcoholism. They discussed the medical framing of alcoholism in the nineteenth and twentieth centuries as ‘slavery of the will’ and ‘a disease of the will’ and inebriation as ‘predisposition’ to alcohol.^86^ Valverde wrote that in using this framework, the medical establishment displayed their incoherence as they could not arrive at a consensus on how to medicalise alcoholism. They could not class alcoholism as separate from ‘the narcomania’ of other drugs nor could they settle on ‘the personality type’ of the addict.^87^ They were also not univocal in their medical critique of the dangerous effects of alcoholism such as ‘evolutionary degeneration’, liver or brain damage.^88^

Addiction scholarship has underlined that alcoholism – initially termed ‘habitual drunkenness’ – evolved through scientific practices of refutation and refinement. In Scottish physician Thomas Trotter’s writing at the dawn of the nineteenth century, drunkenness was socially transmitted but featured as a pathology of mental habit.^89^ An American physician writing around the same time Benjamin Rush had a similar explanation about chronic spirit consumption giving rise to the ‘disease of habituation’.^90^ This gave way to sharper and more specific formulations by the early nineteenth century French psychiatrist Jean-Etienne Esquirol, such as ‘dipsomania’ or ‘drinking in excess’ and monomania or ‘compulsive drinking’, which arose from ‘partial insanity’.^91^ James Nicholls wrote that a Swedish physician, Magnus Huss, deployed terms like ‘chronic’ and ‘acute alcoholism’ to imply ‘constant drinking’ and ‘bouts of extreme intoxication’ and linked alcoholism to the nervous system. Thomas Sutton, an English physician argued in 1813 that ‘delirium tremens’ borne of excessive drinking was a disease in and of itself and an ‘affection of the brain’.^92^

Compared to this, Telugu missionary publics were much more united in their critique of ‘alcoholism’ even if they too did not have a clear definition of the term. They found certain dietetic arguments against alcoholism to be more persuasive than others. *Vivekavathi* consistently argued against alcoholism on grounds of poor digestion, bad nutrition and toxicity. They argued that it caused lasting harm to the stomach and digestive fluids, the liver, heart and nervous system. While they did mention the brain and discuss insanity occasionally, they did not classify them much. They were selective in accepting dietetic arguments and overlooking deeply technical psychiatric arguments of dipsomania, monomania and delirium tremens within texts authored by physicians such as Victor Horsley and Mary Sturge, Henry Munroe and William Hargreaves, whom they cited. This could be because *Vivekavathi* authors found dietetic and generic neurological arguments to be socio-culturally familiar while they read clinical psychiatric definitions to be alienating.

Scholarship on medical temperance in India has not tended to alcoholism *per se* but has subsumed this question across historiographical discussions of subaltern, labour, military history and cultural studies. David Hardiman’s work has been pioneering in that he situated drink scholarship within the Subaltern Studies tradition of foregrounding peasant and Adivasi worlds.^93^ His work and that of Indira Munshi Saldanha have demonstrated toddy and mahua arrack to inhabit Adivasi worlds as a customary, ritualistic, dietary, medicinal and healing practice. These drinks formed the centrepiece of tribal insurgencies against colonial excise regimes, ‘landlords, usurers, and liquor dealers’.^94^ In his more recent work, Hardiman also documented the work undertaken by missionaries to de-couple Christianity from liquor. Alongside providing medical missions to help Adivasis and Dalits, they wished to enforce vows of abstinence and temperance norms. Hardiman’s scholarship, while deploying ethnographic vernacular resources, is limited by an English language colonial and missionary archive. An engagement with the vernacular archive is replete with possibilities of discerning complex narratives of caste and nuanced overlaps between the languages of the medical and the spiritual, the empirical and the didactic and the regional and the global.

Historian Darinee Alagirisamy explained the political saliency of foregrounding *nira* or unfermented toddy as a healthy drink in colonial Madras Presidency.^95^ She wrote that the Tamil Nadu Congress strove hard to garner acceptance for *nira* as both indigenous and nutritionally superior to tea and coffee. David Fahey and Padma Manian outlined the international influences of the Victorian vegetarian movement and Gandhi’s encounter with Liberal temperance advocates in England and South Africa on his nationalist discourse driven by ‘self-purification’.^96^ Harald-Fischer Tine presented the internal class biases about alcohol within British colonial society where it was deemed imperative to police the access of soldiers and sailors or the ‘white underclass’ to arrack for medical and racial reasons.^97^ Lucy Carroll and Eric Robert Colvard traced the deep-seated affinities of missionary and nationalist temperance work across India, the UK and America. This was evident when Congress workers and Anglo-Indian Temperance Association – an organization in India with both British and Indian temperance workers – strategised collaboratively.^98^ They also documented the influence that doctors wielded in shared and separate forums and medical arguments about paralysis, stomach disorders and delirium tremens that featured in their temperance work. While all this work speaks in passing to medical questions of drinking, this article is distinctive in that it foregrounds caste as a structuring principle of discussions of alcoholism. Barring Lucy Carroll and Darinee Alagirisamy’s work, this scholarship on alcohol and temperance in India does not systematically approach alcoholism as a historical reflection on caste. It also does not address itself to Indian language-based archival sources.

Scholarship on race, caste and slavery have illuminated the field of medical evolution. Samuel Roberts argues that the historical progression of diseases like tuberculosis and AIDS can illuminate structures of ‘racial utilitarianism’ or racialised practices and logics of responding to urban industrial capitalism.^99^ Jim Downs’ (2021) work outlines how the bodily degradation of the black slave in the slaveship, plantation and battlefield carved out concrete opportunities for universal theories about overcrowding, smallpox and yellow fever. ^100^ Similar and yet different in its social framing, scholarship in India has commented on the role of caste in determining medical practices. Kancha Ilaiah Shephard, David Arnold and Sohini Bhattacharya have demonstrated how medical science in late nineteenth century colonial India deeply benefited from enslaving caste labour and stigma. Shephard points to the historical trend of relying on Dalit labour to carry out graveyard work during the bubonic plague, as this was work that no one would perform.^101^ Arnold wrote that colonial post mortem practices of dissection evolved through the recruitment of a Dalit community of Doms who alone were willing, where all other castes refused, to assist medical practitioners in this practice.^102^ Sohini Chattopadhyay described the sanitary science of mortuaries to have drawn on the Dalit labour of Mahars and Doms all the more during colonial outbreaks of cholera and plague.^103^ This article synthesises these insights from the social history of medicine with those of Drink and Dalit Studies to delineate alcoholism as socio-medical enslavement within the context of the early-twentieth-century Telugu print sphere.

## Disaggregating languages of medical temperance

Unlike *Vivekavathi*, the two other Telugu journals, *The Telugu Baptist* and *UCH*, decried alcohol itself rather than toddy and arrack specifically. Their usage of the term ‘enslavement’ did not index counter-caste voices in that their discussions lacked local descriptions of caste inequality, landlordism, debt and stigma. What this also implies is that not all universal narratives of medical temperance carried the promise of counter-caste assertion.

These two journals provided Christian instruction for a clean and healthy body expressed in medical terms. They too referred to alcohol causing adverse physiological outcomes, but they deployed a less hybrid language than *Vivekavathi.* Their medical dictums were embedded in long scriptural exposition mainly from St Paul’s Epistles and the Old Testament. Unlike *Vivekavathi*, the medical expositions in these journals shied away from commenting on the local power dynamics of alcohol. They took a more casual approach to all kinds of alcohol as bad, with articles seeking the separation of believers not so much from toddy and arrack as from all intoxicants like opium, cannabis and alcohol.

The term ‘slave’ features here too, but drinkers were presented as slaves to *swadesha, videsha sarayilu* [local and foreign alcohols] rather than *kallu* and *sara.* These journals re-published articles that featured in *Vivekavathi* and, in so doing, shared biases against toddy and arrack. However, they did not feature any original content that carried potent and unveiled messages against *kallu* and *sara*, in the name of temperance.

While medical temperance campaigns found several vehicles, such as catechisms, hymns and experiments in *Vivekavathi*, the other two journals preferred scriptural commentary. Sometimes, these entailed the invocation of one denomination as superior to another in terms of their internal spiritual strictures against alcohol.^104^ Where medical analogies were offered in these journals, they were always justified in Biblical terms, compared to *Vivekavathi*, where the Biblical was expressed often as a matter of form rather than argument. *Vivekavathi* used various Christian devices but did not cite scripture very much and sometimes omitted all allusions to Christianity in providing health insights.

These journals, on the other hand, turned resolutely to the Old Testament as well as verses from St Paul’s Epistles – to warn believers against slavery to alcohol. One article categorically stated that alcohol disrupts blood flow and impacts liver function, burns the heart and decreases life expectancy, and it rapidly turned to the Book of Proverbs to warn,

Be not among winebibbers; among riotous eaters of flesh: For the drunkard and the glutton shall come to poverty: and drowsiness shall clothe a man with rags (Proverbs 23: 20–21).^105^

The message of slavery and the need to escape it is more explicit in passages such as this:

If it is a yes, is it right to make impure our body with tobacco, opium, weed, and alcohols where the almighty God, our father, would like to reside? So, as Christians, ‘Stand fast therefore in the liberty wherewith Christ hath made us free, and be not shackled again by the yoke of slavery’ (Galatians, 5: 1). As said here, we should not touch, taste or be enslaved by these poisonous substances and seek help from the Holy Father to help others from this plight.^106^

*The Telugu Baptist* conveyed bodily suffering in medical terms and pinpointed disease as the manifestation of a failure to recognise that the body is ‘the temple of God’, not to be corrupted with alcohol. Here, it is proclaimed that

If any man defiles the temple of God, him shall God destroy; for the temple of God is holy, which temple ye are’ (1 Corinthians 3: 17).^107^

While strictures against wine-bibbers and riotous eaters of the flesh may be an allusion to the stigmas that caste presents – given that Dalits and other marginalised castes were vilified as beef-eaters and alcoholics – in the absence of context, this reference can neither be regarded as reinforcing or resisting this stigma. These scriptural commentaries did not amount to a call to end slavery among Dalits through the agency of temperance as much as an exhortation to Telugu society, a subset of Indian society, to embrace good habits such as forgoing both tobacco and alcohol. For only a ‘clean body’ and one that is purged of *durvyaparamu* or ‘dissipation’, which results from drinking wine (a placeholder for all alcohol), in the estimation of St Paul^108^ could be the ideal abode of God.

These journals placed alcohol’s physical afflictions side by side with those wrought by tobacco and opium, but they saw alcohol as way more dangerous. *The Telugu Baptist* made references to *nallamandu* or ‘opium’ addiction in China, where the dependence on the poppy seed derivate grew unchecked: ‘the people of China start by imbibing opium doses of as little as a green lentil, but soon, this increases to consuming opium in dosages that resemble a raw egg’.^109^ The consequences of opium addiction were explained to be day-time sleepiness, nausea, bodily lethargy, impaired mental faculties and poverty. The author regards tobacco consumption to be both unchristian, in that it involved squandering money that could go to the Church, and lethal, in that it impaired speaking, hearing and seeing faculties.^110^ But smoking was dangerous in that it represented a sliding scale of slipping into alcoholism. Alcoholism, however, outpaced the unmitigated health disasters of smoking tobacco and opium in that it dramatically reduced life expectancy:

They taste the local and foreign alcohols first, feel a growing attachment the second time, and *become slaves to alcohol* the third time. Compared to the other intoxicants we have discussed above, alcohol is much more dangerous. It disrupts the flow of the blood, impacts liver function, burns the heart, and kills you a little day by day. It decreases your life expectancy. People die suddenly because of their drinking habits (emphasis mine).^111^

The alcoholic body, marked by the failing heart and liver and diminished blood circulation, was abstracted from a social entity and suffered the same physical consequences everywhere. Enslavement here was nothing more than alcoholic and more generally, narcotic addiction. In another article, the author has dual advice for students and teachers. While steering clear of enslavement to alcohol, teachers should educate students about alcohol’s harmful effects by conducting scientific experiments to prove this. Students must read ‘Ayurvedic and medical texts’ to educate themselves similarly. ^112^

*UCH* carried calls for the need to educate people in the global gains vis-à-vis temperance, for instance, in Persia and Glasgow. It exhorted readers to use scientific and religious texts to disseminate temperance ideas. The immersion of alcohol within a global landscape of temperance points to the use of universals that shied away from critiquing local power dynamics.^113^

## Conclusion

While Alcohol Studies have examined the place of temperance in social movements in India and elsewhere, they have not studied this systematically within either medical debates or the fine print of vernacular language press and journals and much less within missionary medical discourses of such journals. And it is this unexplored niche of medical writing on alcohol and temperance in reference to caste in Telugu missionary journals of the early twentieth century that this article dwells on.

The form of medical temperance was carefully curated to convey a range of missionary lessons about alcohol’s addictive qualities, toxicity, lack of hygiene, impact on digestion and poor nutritional content. Owing to its desire to present itself as ‘secular’ unlike Christian missionary and Hindu women’s journals of its time, *Vivekavathi* at different points, carried medical pronouncements against alcohol that did not foray into the spiritual realm. On other occasions, it presented medical temperance by using spiritual aids such as allegories, catechisms and hymns. The journal invoked a wealth of forms of ‘the universal’. The universal was present either in terms of Christian narrative devices or medical paradigms of experiments, with its attendant language of observation, results and inferences, or in a synchronised overlap of both. It was also manifest in *Vivekavathi*’s close affinities to British and American medical and missionary publications and the journal’s inclination to use the same narrative genres. But the hybrid form of *Vivekavathi* did not only reflect the distillation of medical with missionary insight but also the appropriation of medical temperance to convey an intense social commentary about caste and caste-coded enslavement to alcohol.

The term ‘enslavement’ – even where it had universal connotations – therefore was differently imbued. Just as it has been shown that there was no single Bible but rather the possibilities of an insurrectionary Bible, abolitionist Bible and pro-slavery Bible^114^, medical texts like Horsley and Sturge’s *Alcohol and the Human Body* as well as Henry Munroe’s *The Physiological Effects of Alcohol* saw multiple interpretations, mutations, mistranslations and selective translations. The socio-medical undercurrents of temperance in certain counter-caste narratives were empowering, while other manifestations were not in *Vivekavathi*, *UCH* and *The Telugu Baptist.*

Similarly, the terms ‘toddy’ and ‘arrack’ too can be placed in a spectrum in terms of their caste connotations in medical discourses. If at times they signalled and performed a crushing stigma, especially where the medical invocation of these drinks was contrived to shame marginalised castes, and Dalits in particular, into renouncing these drinks, not all medical references to them carried out the same discursive functions. Historically, Dalit writing very deliberately synthesised cutting-edge medical findings about alcohol into their own temperance expositions, in a quest for collective self-assertion. The counter-caste voices of *Vivekavathi* spoke in a similar mould to liberate marginalised castes from these stigmas, while preserving the stigma of these drinks *per se.*

## Data Availability

Data supporting this study are not publicly available due to archival policies in Indian states.

## Footnotes

1
*‘Madhyasaramu* No. 6’, *Vivekavathi*, 1, 8 (1910), 272–3, Andhra Pradesh State Archives (hereafter APSA).

2

O. David , ‘Keertana – Temperance lyric’, Vivekavathi 9, 12 (1918), 284 .

3 Editorial titled ‘Nallamandu Pogaku’, *The Telugu Baptist*, 38, 4 (1914), 49–52: 49, APSA.

4 A.C. Mukherji, ‘A Report on the System of Abkari Administration with Respect to Taxation of Toddy in the Presidency of Madras’ (Calcutta: Bengal Secretariat Press, 1895), 2.

5 For a critique of temperance within the Tamil nationalist framework, see Darinee Alagirisamy, ‘The Problem with Neera: The (Un)Making of a National Drink in Late Colonial India’, *Indian Economic and Social History Review*, 2019. Robert Colvard and Lucy Carroll describe the close encounter of the nationalist and missionary worlds of temperance: Robert Eric Colvard, ‘A World without Drink: Temperance in Modern India 1880-1940’ (unpublished PhD thesis: University of Iowa, 2013); Lucy Carroll, ‘The Temperance Movement in India: Politics and Social Reform’, *Modern Asian Studies*, 10, 3 (1976), 417–47. The subaltern framework is explored in David Hardiman, ‘From Custom to Crime: The Politics of Drinking in Colonial South Gujarat’, in Ranajit Guha (ed.), *Subaltern Studies IV: Writings on South Asian History and Society* (New Delhi: OUP, 1985); Indra Munshi Saldanha ‘On Drinking and Drunkenness: History of Liquor in Colonial India’, *Economic and Political Weekly*, 30, 37 (1995), 2323–31.

6 My use of this term is inspired by descriptions of Enlightenment ideals, particularly those of Galileo, who favoured ‘proved scientific truths over literal scriptural meaning’ in that they were grounded in sense experience–derived ‘physical conclusions.’ Maurice A. Finocchiaro, *On Trial for Reason: Science, Religion and Culture in the Galileo Affair* (Oxford: OUP, 2019), 103.

7 Gareth Atkins, Shinjini Das and Brian H. Murray, ‘Introduction’, in *Chosen Peoples: The Bible, Race and Empire in the Long Nineteenth Century* (Manchester: Manchester University Press, 2020), 1.

8 I found files in United Theological College, Bangalore, containing catalogues of CLS temperance publications in Hindi, Telugu, Tamil and Urdu.

9 This community is today counted as ‘Other Backward Class’ in the list published by the Government of Tamil Nadu.

10

Y.G. Bonnell , The Book I: The Autobiography of YG Bonnell (Madras: Kesari Printing Works, 1930), India Office Records, British Library, London.

11

Y.G. Bonnell , Kutumba Matuvilakku Sastiram (Madras: Thompson and Co., 1916), 4, File No. 105094, Roja Muthiah Research Library Archives, Chennai, see 20–32.

12
*Ibid.,* 4.

13 A brief discussion can be found in Colvard, *op. cit.* (note 5) and Ian Tyrrell, *Woman’s World/Woman’s Empire: The Woman’s Christian Temperance Union in International Perspective 1880-1930* (Chapel Hill, NC: University of North Carolina Press, 2010).

14 Shaik Mahaboob Basha, ‘Christian Press and Hindu Social Reform: The Story of *Vivekavathi*’, *Kakatiya Journal of Historical Studies* (2021), 114–135, 127.

15 Chandra Sekhar writes that the missions that came together to produce *Vivekavathi* were the Church Missionary Society, London Missionary Society, Canadian Baptist Missionary Society, Andhra Evangelical Lutheran Mission, American Baptist Mission, Ceylon and India General Mission. Chakali Chandra Sekhar ‘Christian Women’s Journals and Vernacular Christianity: A Case Study of Vivekavathi in Telugu-Speaking Regions’, *South Asia: Journal of South Asian Studies* (2023), DOI: 10.1080/00856401.2023.2218237.

16 Basha, *op. cit.* (note 14), 118.

17

Elizabeth McCauley , ‘Christian Literature in India’, Lutheran Woman’s Work, 12, 8 (1919), 254 . I thank Chakali Chandra Sekhar for sharing this resource with me.

18
*Ibid.,* 254.

19 David Arnold, *The Congress in Tamilnad: Nationalist Politics in South India 1919-37* (London and New York: Routledge, 1977), 65.

20 Tyrrell, *op. cit.* (note 13), xii, 64, 187.

21 The Band of Hope was a term used to refer to British children’s temperance organizations; it had its roots in British Sunday school in the nineteenth century.

22 ‘Sampadakulu’, *Vivekavathi*, 1, 1 (1909), 1–7.

23 Paik, *op. cit.* (note 6), 72.

24 Mariana Valverde explains that physiologists and psychiatrists in the early nineteenth century construed alcoholism as a disease that incapacitated the will. She argues that this was a poorly medicalised argument. Mariana Valverde , Diseases of the Will: Alcohol and Dilemmas of the Will (Cambridge: Cambridge University Press, 1998).

25

O. David , Mitanubhava Yodhulu, Vivekavathi, 9, 12 (1918), 283 .

26 All three journals were easily available in the Andhra Pradesh State Archives in its older location in Hyderabad in Telangana state. These archives have since moved to Mangalagiri in Andhra Pradesh.

27 C. Rajagopalachari, ‘Experts’ Verdict: Vitamin in Toddy’, *The Hindu*, 5 July 1931, Nehru Memorial and Museum Library, New Delhi.

28 Press clipping, newspaper unknown, 18 March, 1931, Rajaji Papers, IVth Instalment, Subject File 11, Nehru Memorial and Museum Library, New Delhi.

29

Edgar Thurston , Castes and Tribes in Southern India, Vol. 2 (Madras: Government Press, 1909), 2–3, 154.

30
*Dr E. Landon, Alcohol: A Menace to India* (Madras: CLS 1918); Rev. A. Moffat, ‘Drink Traffic in the Madras Presidency’, *Madras Christian College Magazine*, July and August (1909), in *Papers on the Temperance Movement in India* (Madras: Methodist Publishing Houses, 1909), Mss Eur F170/1, *IOR.*

31 ‘Oka Matthuvasthuvukubadulu Inkokati’, *Andhra Harijan*, 2 (30), 3 November 1947; Gajanana Nayak Vyasapangraham, ‘Ekkuva Thatichetlu Veyuta’, *Andhra Harijan*, volume unknown, issue number 29, 14 October 1946, APSA. ‘Harijan’ is a Gandhian term used to refer to Dalits and is regarded to be dehumanizing.

32 Midwives are associated with the marginalised castes, in that their caste labour is devalued, and rickshaw pullers, owing to the nature of their labour and poverty, also conjure images of marginalised castes. Pastor R. Satya Murthy, ‘Madyamuyokka Manchi Cheddalu’, *Arogya Prakashika*, 11, 4, 323, (August 1926) APSA.

33 Anandaswamy, ‘Tragudu’, *Arogya Prakashika*, 7, 2 (1931), 285–7.

34 Basha, *op. cit.* (note 4), 114–35: 124.

35 ‘Patrika Sampadakurali Vyasamu’, *Vivekavathi*, 1, 12 (1918), 255–7: 256. Malas and Madigas are Dalit communities, but the usage of the term is disparaging; there have been movements historically among Dalits to distance themselves from these names, and, in the early twentieth century, the term ‘temperance pledges’ figures either as *vagdattamu* or *pramana patramu* in *Vivekavathi.* It is also key to note that the language of temperance pledges is starkly present in the WCTU and Band of Hope literature.

36
*Ibid.*

37 This tendency was common to the medical temperance narratives in physicians’ writings and in British missionary periodicals such as *The White Ribbon, Alliance News* and *The Rechabite Magazine.*

38 ‘Patrika Sampadakurali Vyasamu’, *op. cit.* (note 35).

39 While explaining the distinctive Indian caste-based aspect of slavery, the historian and Dalit Studies scholar, Chinnaiah Jangam points out that missionaries used to carry pamphlets on *vettichakiri* to ‘every untouchable hamlet in Telangana’. Chinnaiah Jangam, *Dalits and the Making of Modern India* (New Delhi: OUP, 2017), 147. This goes to show the differentiated functions that missionary publications fulfilled.

40 ‘India Deshamuloni Tragudu’, *Vivekavathi*, 7, 9 (1916), 273. While *Vivekavathi* carried the author’s name for certain articles, it omits doing so for others.

41
*Ibid.*

42 ‘*Madyasaramu No. 6*’, *op. cit.* (note 1), 272–3. Experiments involving beef and alcohol were common in medical texts authored by American and British physicians and chemists. An extremely influential medical text on alcohol by Horsley and Sturge contains references to a similar experiment with beef, but here, brandy, whisky, gin and water figure instead of toddy and arrack. Sir Victor Horsley and Mary D. Sturge, *Alcohol and the Human Body: An Introduction to the Study of the Subject* (London: Macmillan and Co., 1907), 211–13.

43 Henry Munroe, *The Physiological Action of Alcohol* (New York: National Temperance Society and Publication House, year possibly 1865); Dr William Hargreaves, *Alcohol and Science: Alcohol, What it is and What it does* (London: William Nicholsons and Sons, year of publication possibly 1875), 110, *Wellcome Library*, London. This Hargreaves version was a $500-prize essay awarded by an American temperance convention. The *Vivekavathi* author would have almost certainly used Dr William Hargreaves’ version, given that a spelling error of Dr Munroe as Dr Monroe occurs in both accounts, and the temperature of the stomach at 100° is consistent to both versions. Besides, a table of findings is presented only in Hargreaves’ version and not in Munroe’s version.

44
*Ibid.* (Munroe).

45
*Ibid.*, 5.

46
*Ibid.*, 5–6.

47

Erica Wald , Vice in the Barracks: Medicine, the Military and the Making of Colonial India (Basingstoke: Palgrave Macmillan, 2014), 129 .

48 Harald Fischer Tine, ‘The Drinking Habits of our Country-men’: European Alcohol Consumption and Colonial Power in British India’, *The Journal of Imperial and Commonwealth History*, 40, 3 (2012), 383–408, 386.

49 This is a bilingually titled article, ‘Alcohol and the Human Body – Madyasaramu’, *Vivekavathi*, 1, 1 (1909), 22–3.

50
*Ibid.,* 23.

51 Horsley and Sturge, *op. cit.* (note 42), 36.

52 K. Satyanarayana, ‘The Dalit Reconfiguration of Modernity: Citizens and Castes in the Telugu Public Sphere’, in Ramnarayan S. Rawat and K. Satyanarayana (eds) *Dalit Studies* (New Delhi: Permanent Black, 2016), 159.

53 Dorothy Porter, *Health, Civilization and the State: A History of Public Health from Ancient to Modern Times* (London and New York: Routledge, 1999, reprint 2005), 63; Roy Porter, *The Greatest Benefit to Mankind: A Medical History of Humanity from Antiquity to the Present* (London: Fontana Press, 1999), 245.

54

Maurice A. Finocchiaro , On Trial for Reason: Science, Religion and Culture in the Galileo Affair (Oxford: OUP, 2019), 103 .

55 Michael Hau, ‘The Dialectic of Medical Enlightenment: War, Alcohol Consumption and Public Hygiene in Germany’, *History*, 104, 359 (2019): 149–68.

56

Sanal Mohan , The Modernity of Slavery: Struggles against Caste Inequality in Colonial Kerala (New Delhi: OUP, 2015), 87–92 , 103.

57
*Ibid.,* 87–92, 103.

58 Jangam, *op .cit.* (note 39), 49–50.

59
*Ibid.,* 51.

60

M. Hariyatamma , ‘Story of a Brahmin and a Dhoby’, Vivekavathi, 6, 9 (1915), 281–3.

61

Nagulaiah , ‘Mitanubhogamu’, Vivekavathi, 6, 9 (1915), 279–80.

62

R.S. Sugirtharajah , The Bible and Empire: Postcolonial Considerations (Cambridge: Cambridge University Press, 2005), 145 .

63 The Third Book of Moses, called Leviticus, 11: 29 makes these strong associations between mice and that which is ‘unclean’; and Nagulaiah’s story forges a fictional connection between unclean mice and *sara.*

64

Sambaiah Gundimeda , Dalit Politics in Contemporary India (New Delhi: Routledge, 2016), 161 .

65 Jangam, *op. cit.* (note 39), 113–4. Dalit and non-Dalit Telugu literature across the twentieth century have reflected on alcohol and debt, albeit with deep differences in register and voice. Among the former, see Arige Ramaswamy, ‘Self-Respect of Savarnas’, in K. Purushotham, Gita Ramaswamy, Gogu Shyamala and Mini Krishnan (eds), *The Oxford India Anthology of Telugu Dalit Writing* (New Delhi: OUP, 2016), 209–14: 210; the poem called ‘The Drunkard Husband’ found in Subbamma, ‘Tagubothu Mogudaye’ in Gogu Shyamala (ed.), *Nallapoddu: Dalita Streela Sahityam 1921-2002* (Hyderabad: Hyderabad Book Trust, 2003), 289–90. Among the latter, see the story called *Maya* in Rachakonda Viswanatha Sastry, *Aaru Sara Kathalu* (place of publication unknown: Visalandhra, 1961). Besides, debt also features in very striking terms as a common theme of oppression against which Adivasi movements (a tribal category of marginalised people) germinated in central India and Bombay Presidency. Saldanha, *op. cit.* (note 5); Hardiman, ‘From Custom to Crime’, *op. cit.* (note 5).

66 Jangam, *op. cit.* (note 39), 114.

67 Gorantla Rangaiah, ‘Matthupadharthamulu’, *Bhagyanagar Patrika*, 15 (1932), 12–13, *Adi-Andhra Bhavan*, Hyderabad.

68 Kusuma Dharmanna, *Madhyapana Nishedhamu* (Vijayawada: Prajashakti, 1930, reprint 2016). ‘The House Beautiful’ analogy features in the *White Ribbon* series and amply in *Vivekavathi* pieces. WCTU author Margaret Denning’s articles in this series were translated from English to Telugu and published in *Vivekavathi.*

69

Pendurti Joseph , ‘Madhupana Nishedha Balabodhini’, Vivekavathi, 11, 1 (1920), 9–10 .

70 A medusa was placed in a jar with fresh water to which one gram of ‘absolute alcohol’ was introduced to ‘a thousand of water’. Another medusa was placed in a jar with just fresh water. It was observed that in the first jar, the medusa’s swimming movements amounted initially to ‘seventy-four in the minute’ but ceased soon until the animal collapsed to the bottom of the vessel. Horsley and Sturge, *op. cit.* (note 42), 56.

71 G. Subramanyam, ‘Tolinati Dalita Shankharavamu’, in Dharmanna*, op. cit* (note 56), 7.

72 Kusuma Dharmanna, *Harijana Shatakamu* (Vijayawada: Prajashakti Bookhouse), 1933, reprint 2016. *Makoddu Ee Nalladoratanamu* (Vijayawada: Prajashakti Bookhouse, 1922, reprint 2016).

73 O. David, ‘Vaidya Vishayamulu: Kshayarogamu Galavaremi Cheyavalenu?’ *Vivekavathi*, 6, 4 (1915), 105–7. This article principally addresses consumptive or tuberculosis patients.

74

O. David , ‘Keertana – Temperance lyric’, Vivekavathi 9, 12 (1918), 284 .

75 O. David, ‘Matanubhava Yodhulu’, *Vivekavathi* 9, 12 (1918), 283; S.B. Gould and A.S. Sullivan, ‘Onward, Christian Soldiers’ in Emmett G. Coleman (ed.), *The Temperance Songbook* (London: Wolfe Publishing, 1974). Songs in this book were ‘rescued’ from the 1907 WCTU archive, Alliance House Foundation Archives, London.

76

O. David , ‘
*Madyapaana Nishedhamu* - Temperance’, Vivekavathi, 9, 12 (1918), 284 .

77
*Ragam* implies a classical musical tune and *talam* means the practice of keeping time within the school known as Carnatic music.

78 Jangam, *op. cit.* (note 39), 113.

79
*Ibid.,* 115–6.

80 He subtly criticises upper caste Hindu activists for taking child marriage seriously but not the *devadasi* system of sexual slavery. O. David, ‘Nizam Rajyamlo Mala, Madiga Sthreela Sthithi’, *Vivekavathi*, 7, 9 (June 1916), 262–3.

81

Urmila Pawar and Meenakshi Moon , We Also Made History: Women in the Ambedkarite Movement (New Delhi: Zubaan Books, 2008), 93–4.

82 O.David, *op. cit.* (note 73), 262.

83

Jessica R. Pliley , Robert Kramm and Harald Fischer-Tine , Global Anti-Vice Activism: Fighting Drinks, Drugs and Immorality (Cambridge: Cambridge University Press, 2016); Nikolay Kamenov , Global Temperance and the Balkans: American missionaries, Swiss scientists and Bulgarian socialists 1870-1940 (Geneva: Palgrave, 2020).

84 Kamenov, *op. cit.* (note 81).

85

Roy Porter , ‘The Drinking Man’s Disease: The Pre-History of Alcoholism in Georgian Britain’, British Journal of Addiction, 80 (1985), 385–96391007210.1111/j.1360-0443.1985.tb03010.x.

86 See Mariana Valverde , ‘Slavery from Within: The Invention of Alcoholism and the Question of Free Will’, Social History, 22, 3 (1997); James Nicholls, *The Politics of Alcohol: A History of the Drink Question* (Manchester: Manchester University Press, 2011), e-book. Also see Valverde, *op. cit.* (note 24).

87 Valverde, *ibid* (‘Slavery from Within’), 257.

88
*Ibid.,* 257.

89

Griffith Edwards , ‘Thomas Trotter’s Essay on Drunkenness Appraised’, Addiction, 107, 9 (2012), 1562–792256391210.1111/j.1360-0443.2012.03890.x, 1564.

90
*Ibid.,* 1565.

91 Nicholls, *op. cit.* (note 85), Porter, ‘The Drinking Man’s Disease’, *op. cit.* (note 84), 390.

92

F.J. Rodriguez Porcel and H.S. Schutta , ‘From Antiquity to the N-Methyl-D-Aspartate Receptor: A History of Delirium Tremens’, Journal of the History of the Neurosciences, 24, 4 (2015), 378–952644492110.1080/0964704X.2015.1034515: 386

93 David Hardiman, *Missionaries and their Medicine: A Christian Modernity for a Tribal India* (Manchester: Manchester University Press, 2017); Hardiman, *op. cit.* (note 5) ‘From Custom to Crime’.

94
*Ibid.,* 21; Hardiman, *op. cit.* (note 5); Saldanha, *op. cit.* (note 5).

95 Darinee Alagirisamy, *op. cit.* (note 5).

96

David Fahey and Padma Manian , ‘Poverty and Purification: The Politics of Gandhi’s Campaign for Prohibition’, The Historian, 67, 3 (2005), 489–506 .

97 Fischer-Tine, *op. cit.* (note 49).

98 Carroll, ‘The Temperance Movement in India’; Colvard, ‘A World without Drink’, *op. cit.* (note 5). Colvard, however, traces dissonances in these affinities in terms of missionaries pushing their own agendas.

99

Samuel Roberts , Infectious Fear: Politics, Disease, and the Health Effects of Segregation (Chapel Hill, NC: University of North Carolina Press, 2009), 5 .

100

Jim Downs , Maladies of Empire: How Colonialism, Slavery and War Transformed Medicine (Cambridge, MA: Harvard University Press, 2021).

101 Kancha Ilaiah Shephard, ‘Practice Disease Distancing – How India Can Use Corona Crissis To kill Its Caste Virus (2020), available at https://theprint.in/opinion/practice-disease-distancing-india-corona-crisis-kill-caste-virus/394162/ (accessed on 15 March 2023).

102

David Arnold , Colonizing the Body: State Medicine and Epidemic Disease in Nineteenth-Century India (Berkeley: University of California Press, 1993).

103 Sohini Chattopadhyay, ‘What Researching Cremations of the Dead in Colonial India Taught Me About Life in Our Cities Today’ (2020), available at https://scroll.in/article/971096/what-researching-cremations-of-the-dead-in-colonial-india-taught-me-about-life-in-our-cities-today (accessed on 10 April 2023).

104 M.E. Archibald, a white editor of *Vivekavathi*, wrote for *The Telugu Baptist* as well. She described how she met a Christian who expressed joy that Baptists took their covenant with God seriously, unlike other denominations, and abhorred all intoxicants, alcohol and cigarettes alike. ‘Total Abstinance (sic) - Madyapaana Nishedhamu’, *The Telugu Baptist*, 35, 10 (1911), 145–9.

105 Editorial titled ‘Nallamandu Pogaku’, *The Telugu Baptist*, 38, 4 (1914,) 49–52.

106
*Ibid.*

107
*Ibid.*

108
*The Telugu Baptist* article carries the warning, ‘Do not get drunk on wine, in which is dissipation. Instead, be filled with the Spirit (The Epistle of Paul the Apostle to the Ephesians 5: 18)’. *Ibid.*

109
*Ibid.,* 50.

110
*Ibid.*

111
*Ibid.,* 49. The Telugu term *lobaduta* is recurringly used; this can be contextually translated into ‘succumbing’ to addiction, or a physical enslavement to alcohol.

112 ‘Upadhyayulanu Vidyarthulanu Madhyapana Nishedhamunaketla Sahapadagalaru - What can Teachers and Students do for Temperance’, *The Telugu Baptist*, 25, 12 (1911), 191–2.

113 ‘Temperance Notes - Madyapaana Visarjana Vartalu’, *The United Church Herald*, 1, 12 (1910), 139–40, APSA.

114 John Coffey, ‘A Bad and Dangerous Book? The Biblical Identity Politics of the Demerera Slave Rebellion’ in Atkins, Das and Murray (eds), *op. cit.* (note 7).

